# Testosterone treatment impacts the intestinal microbiome of transgender individuals

**DOI:** 10.1128/msphere.00557-24

**Published:** 2024-09-10

**Authors:** Rebecca M. Harris, Fernanda Pace, Thomas M. Kuntz, Xochitl C. Morgan, Phoebe Hyland, Kiana Summers, Em McDermott, Kai Blumen, Paula I. Watnick

**Affiliations:** 1 Division of Endocrinology, Boston Children’s Hospital, Boston, Massachusetts, USA; 2 Department of Pediatrics, Harvard Medical School, Boston, Massachusetts, USA; 3 Division of Infectious Diseases, Boston Children’s Hospital, Boston, Massachusetts, USA; 4 Harvard Chan Microbiome Analysis Core, Department of Biostatistics, Harvard Chan School of Public Health, Boston, Massachusetts, USA; University of Michigan-Ann Arbor, Ann Arbor, Michigan, USA

**Keywords:** gut microbiome, human microbiome, testosterone, arginine metabolism, metagenomics, transgender

## Abstract

Medical modulation of sex hormone levels is a cornerstone of treatment for many conditions that impact well-being, including cancer, fertility/infertility, gender dysphoria, and chronic metabolic diseases such as diabetes and obesity. The microbial residents of the intestine, known as the microbiota, interact with sex hormones in the intestine, and there is correlative evidence that this interaction is bidirectional. Based on these published findings, we hypothesized that transgender individuals receiving exogenous testosterone as part of their gender-affirming medical treatment might undergo changes in their intestinal microbiome. To test this, we collected 26 stool samples from nine individuals before and up to 8 months after initiation of treatment with exogenous testosterone and subjected these samples to metagenomic analysis. While no species were significantly associated with the duration of testosterone therapy, pathways that generate glutamate increased in abundance, while those that consume glutamate decreased. Glutamate is a precursor of arginine, and testosterone is known to increase levels of arginine and its metabolites in the plasma. We hypothesize that testosterone increases the uptake of glutamate by enterocytes, thus decreasing access of the microbiota to this amino acid. While this pilot study establishes the impact of testosterone therapy on the intestinal microbiome, a more comprehensive study is necessary to establish the impact of testosterone-driven metagenomic shifts on the stool metatranscriptome, the stool metabolome, and the plasma metabolome.

**IMPORTANCE:**

The human intestine is inhabited by a large community of microbes known as the microbiome. Members of the microbiome consume the diet along with their human host. Thus, the metabolomes of the host and microbe are intricately linked. Testosterone alters the plasma metabolome. In particular, plasma levels of arginine and its metabolites and testosterone are positively correlated. To investigate the impact of exogenous testosterone on the microbiome, we analyzed the stool metagenomes of transgender individuals before and after the initiation of testosterone treatment. In this pilot project, we found a modest impact on the microbiome community structure but an increase in the abundance of metabolic pathways that generate glutamate and spare glutamate consumption. We propose that the host uses glutamate to generate arginine, decreasing the amount available for the microbiome.

## INTRODUCTION

An individual’s predisposition to chronic metabolic diseases, such as type 2 diabetes mellitus or obesity, depends on many interdependent factors including host genetics, biological sex, plasma sex hormone levels, and the composition of the intestinal microbiota (1–16). Here, we describe a pilot study to explore how testosterone impacts the intestinal microbiota when host genetics and biological sex remain constant.

The rationale for this study is founded on the known interaction between sex hormones and the intestinal microbiota. Like bile acids, sex hormones are synthesized from cholesterol and undergo enterohepatic circulation in which they are conjugated in the liver, excreted into the intestinal lumen, deconjugated and modified by the intestinal microbiota, and reabsorbed into the hepatic circulation in their deconjugated, modified form (10, 17, 18). Microbes that derive nutrients or energy from sex hormone deconjugation and/or modification are likely advantaged within the intestinal microbial community. They, in turn, alter plasma levels of sex hormones and may secrete other metabolites that are detected in the systemic circulation (8, 18). In fact, sex hormones have been shown to alter plasma metabolites, and a correlation between the plasma and stool metabolomes has been demonstrated (19–21).

Based on these observations, we hypothesized that testosterone administration might favor members of the intestinal microbiome with particular metabolic traits. To test this, we carried out a prospective study to explore how the administration of testosterone impacts the intestinal metagenome of transgender individuals. No significant associations were found between testosterone administration and community complexity, nor with the relative abundances of any individual species, organized as species-level genomic bins (SGBs). However, testosterone administration was associated with modest shifts in the overall community structure, as well as significant shifts in the abundance of genes encoding enzymes involved in lipid, carbohydrate, nucleic acid, protein, and cell wall catabolism. Glutamate is a precursor of arginine. Notably, the abundance of genes in the arginine biosynthesis pathway as well as other pathways that generate glutamate increased, while those that consume glutamate decreased. Plasma concentrations of arginine and its metabolites are higher in cisgender men than cisgender women and increase with the administration of testosterone to transgender men (19, 22–24). We propose that testosterone increases host absorption of glutamate from the diet, favoring microbes that utilize glutamate-sparing metabolic pathways.

## RESULTS

### Study design, recruitment, and sample collection

We recruited nine transgender individuals between the ages of 14 and 20 prior to starting exogenous testosterone as part of their gender-affirming medical treatment. Each individual collected stool samples prior to and up to two times after the initiation of testosterone. The stool was immediately stored in the patient’s home freezer and then delivered to the clinic for storage at −80°C. Stool samples for DNA isolation and calprotectin measurements were collected in separate vials. If both samples were collected and received by the research team within 3 days, one vial was submitted for calprotectin quantification. Demographic data for the individuals and stool collection times are shown in Table 1.

**TABLE 1:** Cohort information^*a*^

Participant ID	Sex assigned at birth	Race^*b*^^,^^*c*^	Hispanic or Latino/e/x^*b*^	Gender identity^*b*^^,^^*c*^	Gonadotropin-releasing hormone agonist treatment prior to testosterone	Pubertal status at testosterone initiation	Adjunct hormonal medications^*d*^	Time on testosterone at each sample (days)
1	Female	Asian, Caucasian	No	Male, transgender male	No	Post-menarche	Drospirenone	S1: 0 S2: 31 S3: 116
2	Female	Caucasian	No	Transgender male	No	Post-menarche	None	S1: 10 S2: 54 S3: 139
3	Female	Caucasian	Yes	Male, transgender male	Yes	Post-menarche	Norethindrone	S1: 0 S2: 53 S3: 114
4	Female	Caucasian	No	Transgender male, non-binary/gender queer/gender fluid	No	Post-menarche	Levonorgestrel IUD	S1: 0 S2: 9 S3: 94
5	Female	Caucasian	No	Male, transgender male	No	Post-menarche	Ethinyl-estradiol and norethindrone	S1: 0 S2: 35 S3: 120
6	Female	Caucasian	No	Transgender male	No	Post-menarche	Norethindrone	S1: 0 S2: 38 S3: 249
7	Female	Caucasian	Yes	Transgender male	Yes	Post-menarche	Norethindrone	S1: 0 S2: 126
8	Female	Caucasian	No	Transgender male	No	Post-menarche	None	S1: 0 S2: 21 S3: 91
9	Female	Caucasian	No	Transgender male	No	Post-menarche	Norethindrone	S1: 0 S2: 37 S3: 132

^*a*^ Race, Hispanic/Latino/a/x status, and gender identity were self-reported, and the remaining information was obtained from the electronic medical record.

^*b*^ Self-reported.

^*c*^ Participants allowed to select >1 race and gender.

^*d*^ Adjunct hormonal medications were initiated prior to starting testosterone and did not change during the course of the study except for participant 5 who was taking combined ethinyl-estradiol and norethindrone when the first stool sample (S1) was obtained and then stopped the medication prior to the second sample (S2).

### Stool calprotectin levels do not change with testosterone therapy

**Fig 1:**
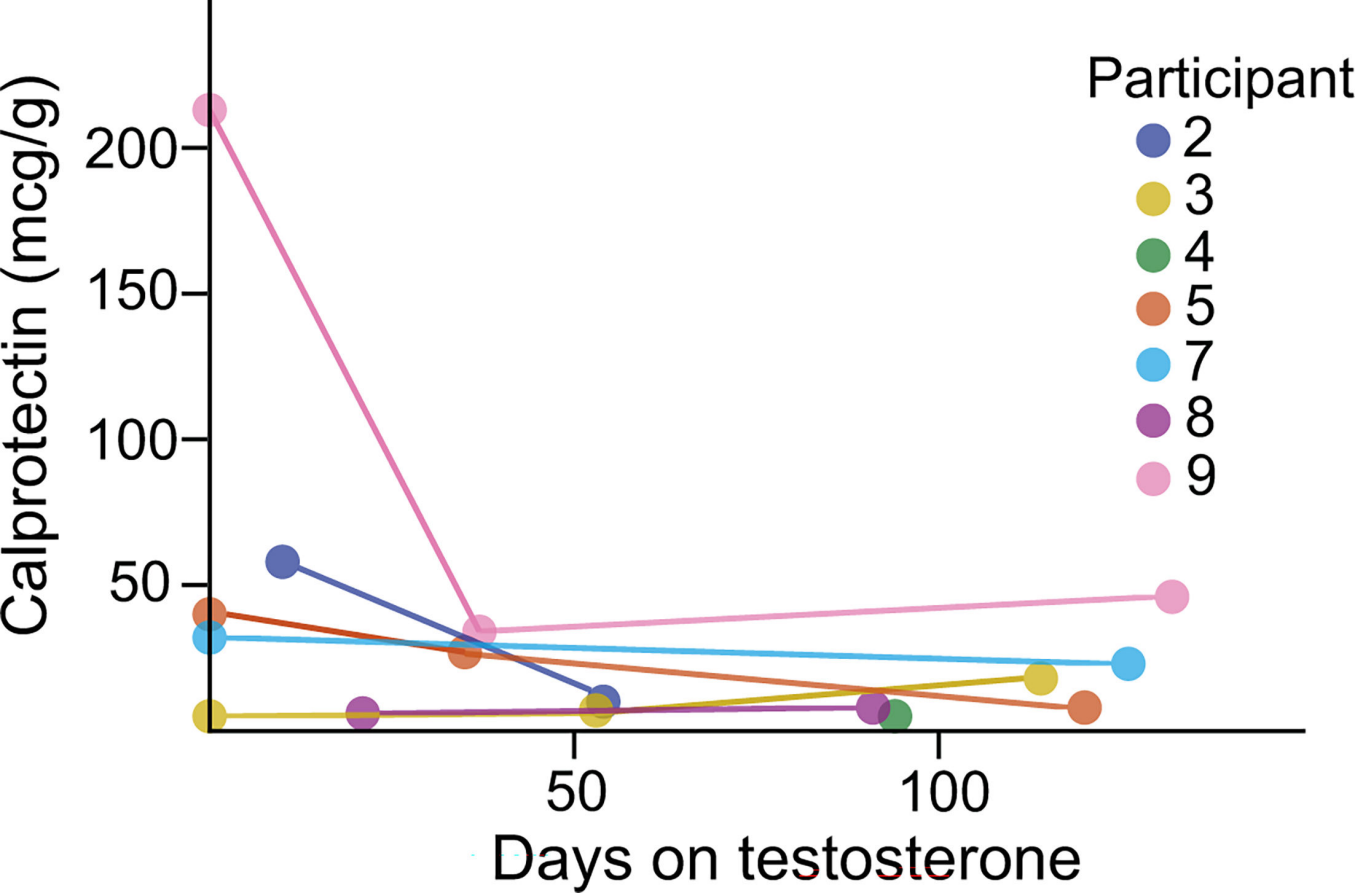
Calprotectin levels did not vary significantly with testosterone administration. Calprotectin levels were measured for stool that was received within 3 days of collection. ^ns^*P* > 0.05.

Inflammation is well known to reshape the microbiome. Furthermore, testosterone can have an anti-inflammatory effect on tissues. To control for intestinal inflammation as a confounding variable, we measured calprotectin levels. While a range of calprotectin levels was measured over the course of the experiment, no trend was observed, suggesting that testosterone treatment does not significantly modulate intestinal inflammation (Fig. 1).

### Species-level microbiome composition is not correlated with the duration of testosterone treatment

**Fig 2:**
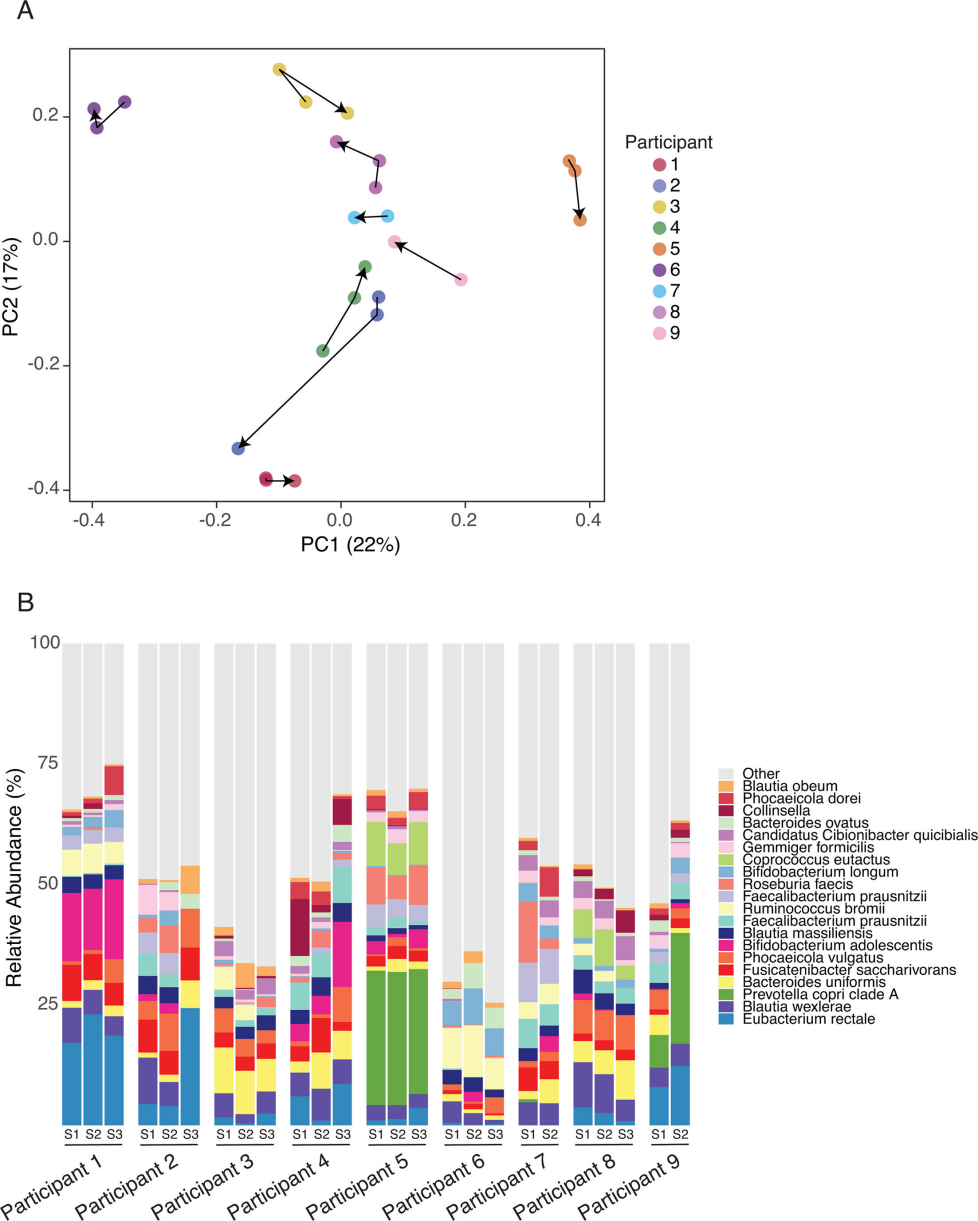
Species-level microbiome composition is not correlated with the duration of testosterone treatment. (**A**) Principle component analysis of individual microbiomes showing clustering in two dimensions of an individual’s microbiome over time. Arrows indicate progression over time for each additional sample. (**B**) Bar plot showing the relative abundance of major genera and species found in the stool of individuals at each of the study time points.

Shotgun metagenomics was performed on DNA isolated from the stool samples collected (Table 1) and used to assess the microbiome diversity within (alpha diversity) and between (beta diversity) individuals over the course of the experiment. Alpha diversity, as measured by the Shannon index, trended downward over time but did not reach statistical significance (coefficient = −0.002, *P* = 0.06) (Table S1). Permutation-based ANOVA revealed a significant relationship between beta diversity, as measured by Bray–Curtis dissimilarity, and testosterone treatment (*R*^2^ = 0.035; *P* = 0.009). The abundance of each species was tested for linear association with time on testosterone, with interindividual differences accounted for by random effects modeling. No associations reached a false discovery rate (FDR) *P*-value cutoff of 0.2 for significance, and the microbiome composition of a fecal sample derived from a given individual remained most like other fecal samples from the same individual rather than like those obtained from other individuals, independent of testosterone administration (Fig. 2). This demonstrates that the effects of testosterone therapy on microbiome composition are modest.

### Significant changes in the functions encoded within the intestinal metagenome occur as a result of testosterone administration

**Fig 3:**
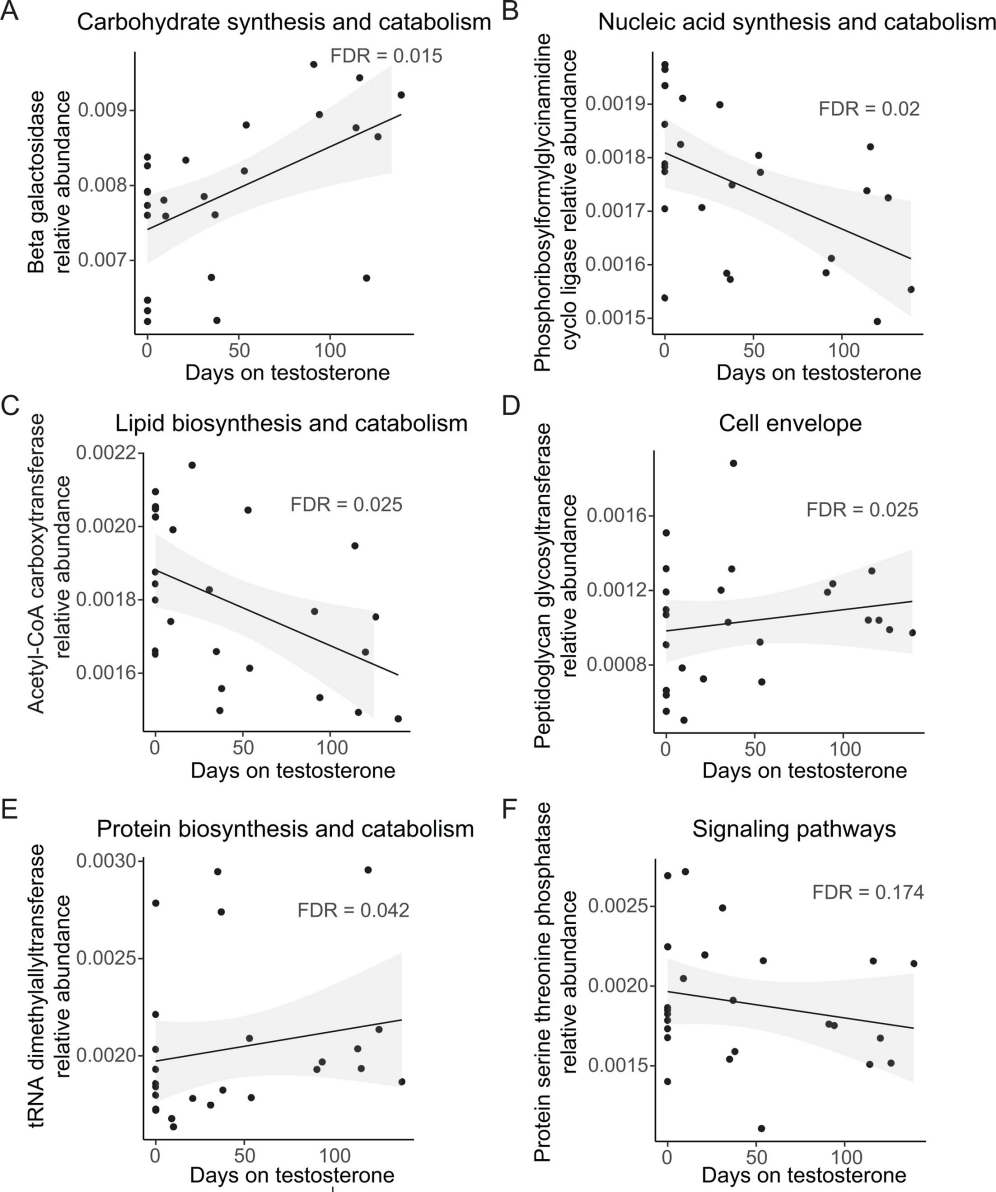
Relative abundance trends after initiation of testosterone for the most significantly modulated genes in each of the major functional classes detected.

**Fig 4:**
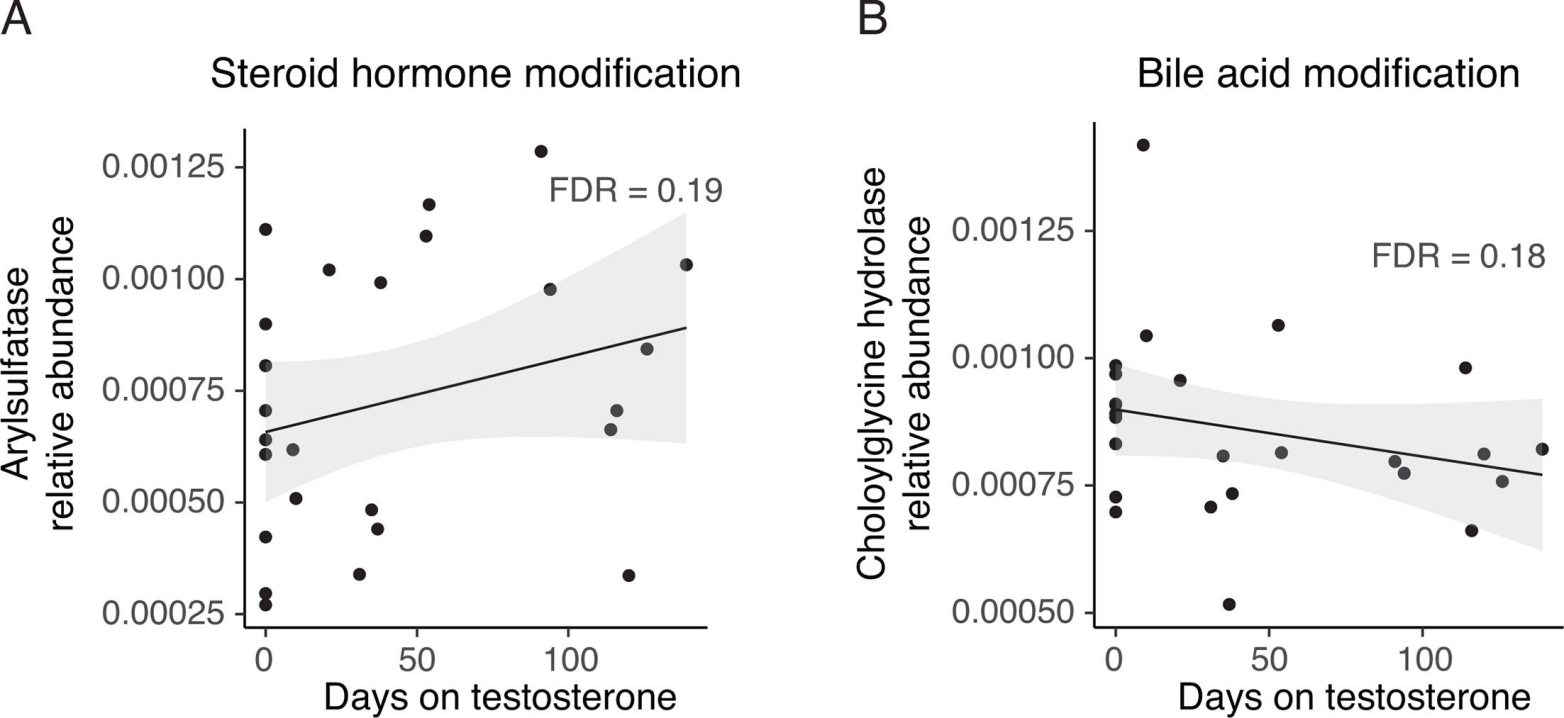
Relative abundance trends for two genes that are predicted to modify a steroid hormone and a bile acid, respectively.

Although we did not detect a shift in the microbiome composition with testosterone administration, we did observe changes in the metabolic potential of the metagenome. Specifically, the abundances of 20 genes were significantly modulated with testosterone administration using a threshold of FDR < 0.05. A less stringent significance threshold of FDR < 0.2 yielded 110 significantly modulated gene abundances. These genes had diverse housekeeping functions including carbohydrate, nucleic acid, lipid, one carbon, cell wall, and amino acid and protein biosynthesis and catabolism (Fig. 3A through E; Table S2). Metagenomic representation of other enzymes known to alter intracellular concentrations of small, regulatory molecules such as diadenylate cyclase, which generates c-di-AMP; GTP diphosphokinase, which generates ppGpp; and cyclic diguanylate-specific phosphodiesterase, which hydrolyzes c-di-GMP, was also changed in response to testosterone administration (Fig. 3F; Table S2). Interestingly, the gene encoding arylsulfatase, which is predicted to modify host steroid hormones, increased with testosterone administration, while that encoding a choloylglycine hydrolase, which is predicted to modify host bile acids, decreased (Fig. 4).

**Fig 5:**
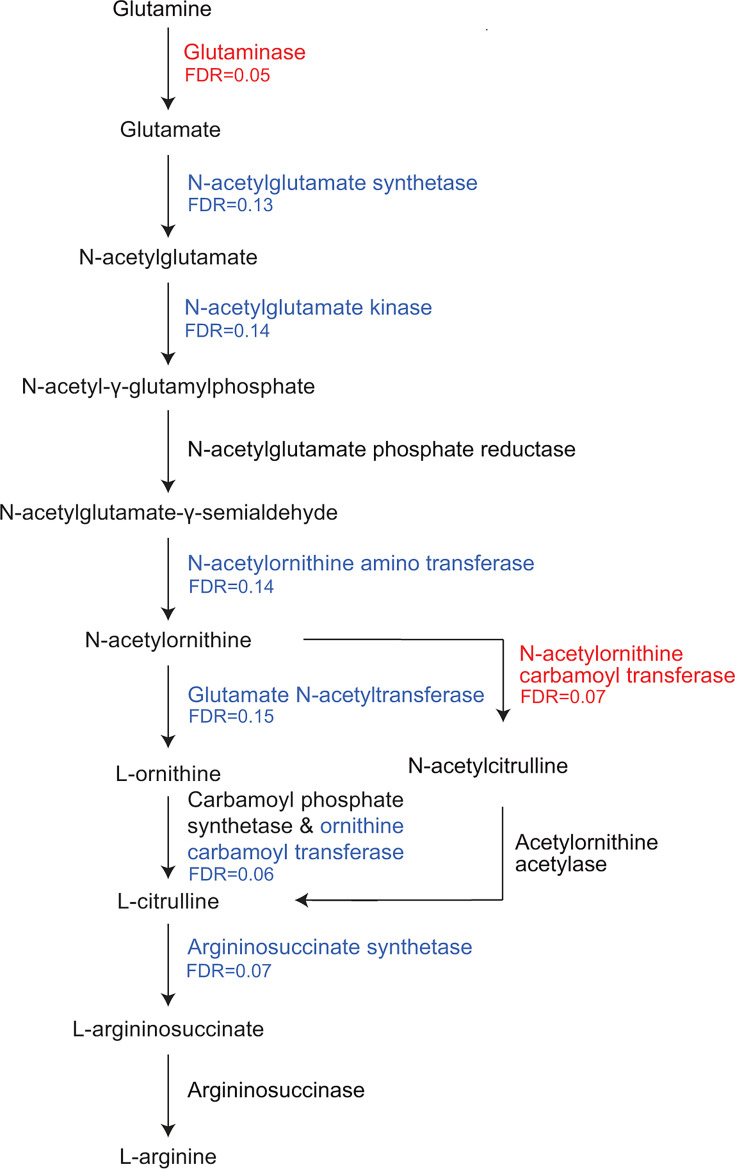
Microbial arginine biosynthesis pathway. Schematic showing genes in the microbial arginine synthesis pathway. Genes whose abundance increases significantly with testosterone treatment are shown in red, while those whose abundance decreases significantly are shown in blue.

**Fig 6:**
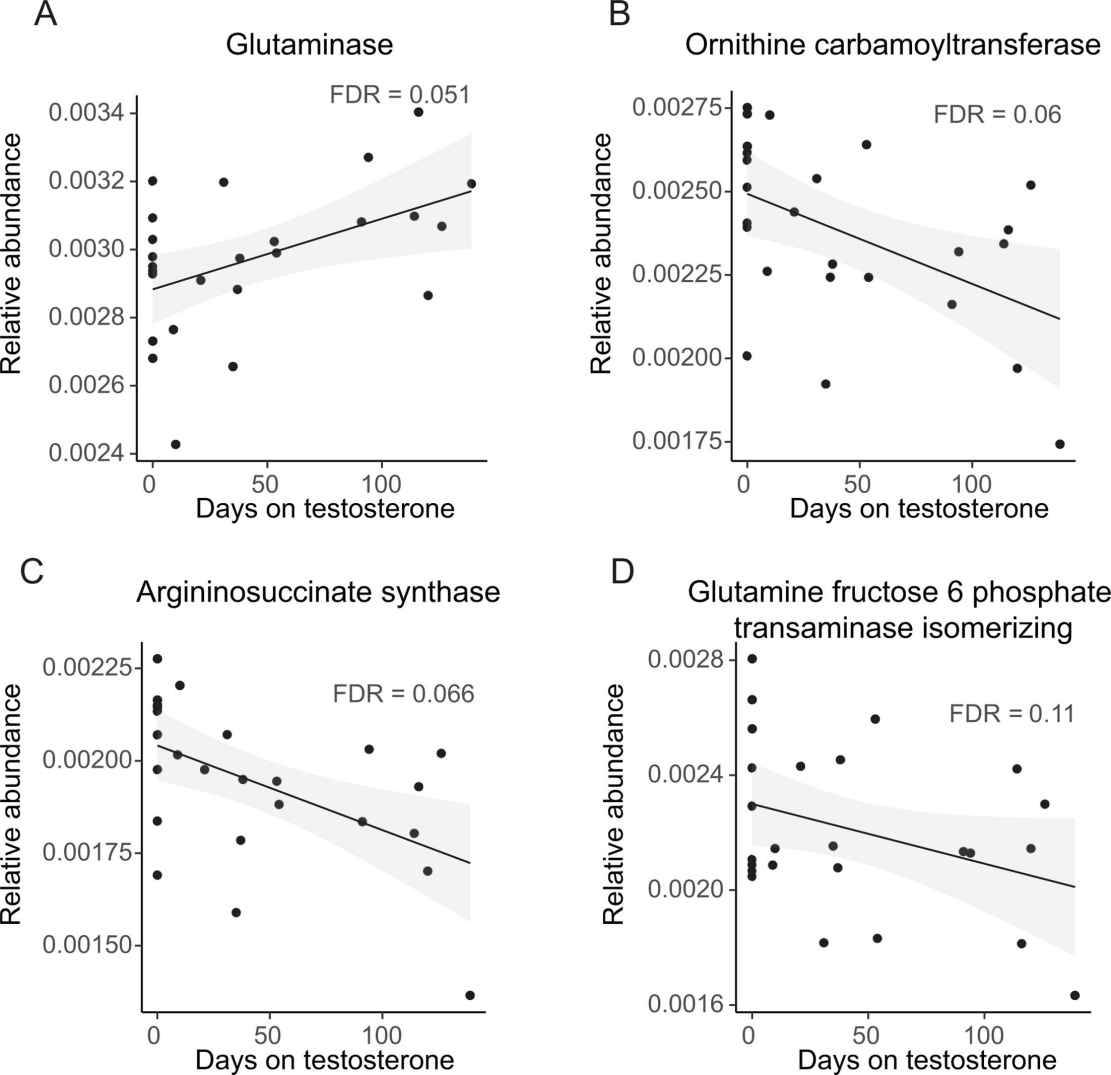
Relative abundance trends of selected arginine metabolic pathway genes with administration of testosterone.

Remarkably, the abundance of nearly all genes encoding enzymes involved in arginine synthesis and catabolism was significantly modulated by testosterone administration (Fig. 5). Among those whose abundances changed most significantly were genes encoding glutaminase and N-acetylornithine carbamoyltransferase, which increased in abundance, and ornithine carbamoyltransferase and arginosuccinate lyase, which decreased in abundance (Fig. 6; Table S2). Glutaminase generates glutamate from glutamine. N-Acetylornithine carbamoyltransferase, glutamate N-acetyltransferase, and ornithine carbamoyltransferase are components of two bacterial pathways that generate citrulline from N-acetylornithine (Fig. 5). Glutamate N-acetyltransferase and ornithine carbamoyltransferase generate L-citrulline from N-acetylornithine through an ornithine intermediate in a reaction that consumes glutamate. In contrast, N-acetylornithine carbamoyltransferase utilizes carbamoylphosphate to convert N-acetylornithine to N-acetylcitrulline and then citrulline. This route circumvents the utilization of glutamate (Fig. 5) (25).

Several more genes encoding enzymes required to generate arginine from glutamate as well as a spermidine/putrescine ABC transport protein decreased in abundance (Fig. 5; Table S2). Polyamines such as spermidine and putrescine are metabolites of arginine that regulate many cellular functions (26). These findings led us to hypothesize that members of the microbiota that augment glutamate pools at the expense of arginine synthesis and metabolism are favored in the intestines of participants who receive testosterone. We reasoned that, if this were the case, additional genes encoding enzymes that consume glutamine or glutamate would decrease in abundance, while those that augment glutamate pools would increase. In fact, the genes encoding glutamine-fructose-6-phosphate transaminase, which converts glutamine to D-glucosamine-6-P, and amidophosphoribosyltransferase, which consumes glutamate in the synthesis of purines, decreased in abundance, while the genes encoding L-aspartate oxidase, which generates the glutamate precursor oxaloacetate, and glutamine-hydrolyzing GMP synthase, which generates glutamate, increased in abundance (Table S2). Taken together, we propose a model for future investigation in which testosterone administration decreases glutamate availability in the gut, possibly due to increased intestinal absorption. This would favor organisms that have the metabolic versatility to eschew reactions that utilize glutamate and glutamine. In addition, organisms that consume glutamate to generate arginine would decrease in abundance under these conditions.

## DISCUSSION

Many studies have reported differences between the microbiomes of males and females, leading to the coining of the phrase microgenderome (8–10, 27–32). Such differences are thought to impact the predisposition to chronic diseases including obesity, diabetes, atherosclerosis, and autoimmunity (12, 16, 27, 33–39). Based on these studies, we undertook a pilot study designed to assess the impact of gender-affirming testosterone treatment on the stool microbiome of transgender individuals. Our results showed that, at least in the short term, the administration of testosterone does not cause a major shift in the composition of the microbiome community. However, many genes became differentially abundant in the metagenomes of individuals after testosterone administration.

Because of its proximity to consumed food and the rich intestinal blood supply, the intestinal microbiota is ideally positioned to aid in the digestion of the host diet and respond to host metabolism (40–42). In fact, there is ample evidence that the plasma and gut metabolomes are correlated (19, 43, 44). For instance, in one recent study of over 8,000 patients, the authors reported that microbial metabolic functions associated with the degradation of amino acids and monosaccharides were most strongly correlated with plasma metabolites (19). Species-specific microbial metabolic functions were significantly negatively correlated with the plasma levels of arginine, glutamine, N-acetylornithine, and urea, while glutamate, N-acetylglutamate, and N-acetylcitrulline were positively correlated. These relationships may reflect the dietary nutrients the host and the microbiota compete for or collaborate to obtain, respectively.

Enterocytes, the most abundant cells in the intestinal epithelium, play an important role in arginine metabolism. They are the principal site of conversion of plasma and diet-derived glutamine, glutamate, and arginine to citrulline. In fact, plasma citrulline levels are often used as a correlate of intestinal mass (45, 46). Citrulline enters the systemic circulation and is converted to arginine and then nitric oxide or urea via the nephric urea cycle (47). Nitric oxide is an important metabolite of arginine, which decreases vascular smooth muscle tone and maintains endothelial homeostasis. Because enterocytes are a primary site of arginine metabolism, a correlation between arginine in the plasma and arginine precursors such as glutamine and glutamate in the intestine might be predicted.

We aimed to place the changes we observed in the stool metagenome of testosterone-treated individuals in the context of what is known in the literature regarding differences in the plasma metabolome of (i) cisgender males and females and (ii) transgender individuals receiving gender-affirming testosterone treatment. In studies comparing cisgender male and female plasma metabolites, arginine, glutamate, and glutamine were found to be significantly higher in males (23, 48, 49). Furthermore, estradiol therapy in post-menopausal females decreased the plasma levels of arginine (50). These studies suggest that testosterone increases and estradiol decreases the levels of arginine in the plasma of cisgender individuals. A study in transgender males treated with testosterone described an increase in plasma arginine and also methylated forms of arginine such as asymmetric dimethylated arginine (ADMA), which inhibits nitric oxide synthase and could increase the risk of cardiovascular disease (22). A more recent study described plasma metabolomics in 20 transgender males and females prior to and 12 months after the initiation of sex hormone treatment (24). This study also found higher plasma levels of ADMA and a higher ratio of citrulline to arginine in participants assigned male at birth prior to therapy as compared with participants assigned female at birth. Therapy with testosterone or estradiol increased or decreased the ratio of citrulline to arginine, respectively. Therefore, independent of sex chromosomes, testosterone levels are correlated with higher plasma levels of arginine and increased generation of citrulline from precursors such as glutamate in the intestine.

In our metagenomic study, the abundance of enzymes that generate glutamate or spare glutamate consumption increased in abundance with testosterone administration, while those that utilize glutamate and glutamine decreased. Based on these findings, we propose a model in which testosterone treatment increases plasma arginine levels preferentially through the uptake of glutamate from the diet, leading to decreased availability for the microbiota. In other words, testosterone increases competition between the host and the intestinal microbiota for dietary glutamate.

There are limitations of this study that should be considered. First, as this was a pilot study, the sample size was small, and we only collected one sample prior to testosterone initiation. Therefore, an individual’s microbiome stability could not be assessed. Second, fecal metatranscriptomic and metabolomic data are required to demonstrate that the changes we identified in the metagenome lead to differences in gene expression and intestinal metabolite abundance. Last, the interpretation of our results in the context of the published data on the plasma metabolome may not be valid as the impact of testosterone on the plasma metabolome of the individuals who participated in this study was not assessed. Future studies will include a larger sample size and analysis of the plasma and stool metabolomes in concert with the stool metagenome and metatranscriptome to yield a more complete picture.

## MATERIALS AND METHODS

### Study population, sample collection, and storage

Between August 2021 and May 2023, we recruited nine individuals from the Boston Children’s Hospital Gender Multispecialty Service clinic as part of an institutional review board-approved study (IRB-P00029637). Individuals were eligible if they were planning to start treatment with exogenous testosterone as part of their gender-affirming medical care. Individuals were excluded if they had a genetic disorder, had taken an antibiotic within the 2 months prior to starting testosterone, were pregnant, or could not provide assent/consent. We obtained written informed consent from all individuals 18 years of age or older; if an individual was a minor, we obtained written consent from a guardian and assent from the participant. Adult participants and the guardians of minor participants completed demographic and hormonal medication questionnaires. Additional clinical information was collected from the electronic medical record.

Seven of the nine individuals were treated with weekly, subcutaneous testosterone enanthate or cypionate injections (25 mg weekly for 4 weeks and then 50 mg weekly with additional titration as needed to maintain testosterone levels within the cisgender adult male reference range). Participant 4 was initially treated with a transdermal testosterone patch (2 mg/24 hours) but after 7 weeks switched to 50-mg subcutaneous testosterone enanthate weekly due to skin irritation, while participant 9 was treated with 10-mg subcutaneous testosterone cypionate weekly with the dose increased by 10 mg every 3 months.

Study participants were given a sample collection kit for at-home stool collection, which included a Raku-Ryu collection cup (Takahashi Keisei Corporation), nitrile gloves, three collection tubes, two waterproof bags, absorbent pads, an ice pack, and a styrofoam container. Two collection cups were empty, while the third contained 10 mL of 95% ethanol with instructions to add stool to the 15-mL mark. After stool collection, study participants were instructed to package the stool in the two waterproof bags with absorbent material in each bag and then to either place the collected samples in their freezer until their next appointment or have the samples transported to our facility by a medical courier. Samples were immediately stored at −80°C upon arrival. Samples that had been stored in the patient’s freezer for 3 days or less were also sent for calprotectin levels.

### DNA purification and sequencing

Stool samples were aliquoted into cryotubes while frozen and were transported to the Broad Research Institute by a medical courier. DNA extraction and sequencing were performed by the Broad Clinical Laboratory. Stool samples were processed using the AllPrep PowerFecal DNA/RNA 96 Kit (Qiagen). DNA libraries were prepared using a Nextera XT library preparation kit (Illumina), and sequencing was performed on a Novaseq 6000 (Illumina). After quality control, the median number of reads per sample was 48,209,387 with a range of 27,426,289–80,627,193.

### Metagenomic analysis

The metagenomic data were processed using the bioBakery meta’omics workflow following the standard pipeline with default parameters (51). In short, this comprises three fundamental steps: data were first passed through KneadData v0.12.0 for read-level quality control; then, taxonomic profiling was performed using MetaPhlAn v4.0.6 (52), database mpa_vOct22_CHOCOPhlAnSGB_202212, and functional potential profiling with HUMAnN v3.7. MetaPhlAn 4 integrates both metagenome-assembled genomes and isolate genomes to define an expanded set of species-level genome bins referred to as species even when not currently assignable taxonomically at the species level. Taxonomic and functional tables were filtered requiring features to have greater than 0.1% relative abundance in over 10%, except in the case of alpha diversity calculations.

Statistical analyses were performed in the R 4.3.0 environment. In particular, the vegan: Community Ecology Package was used for ecological diversity measurements, tests, and visualizations, and MaAsLin2 was used for the linear modeling framework (53, 54). Among each of the comparison types generated, multiple comparisons are adjusted using a Benjamini and Hochberg procedure using the p.adjust function in R, and FDR-adjusted *P*-values of 0.2 or lower are reported as significant. Where noted, abundance refers to the relative abundance within the metagenome.

## Data Availability

The sequencing data for this project are available as BioProject PRJNA1130508.

## SUPPLEMENTAL MATERIAL

Table S1 msphere.00557-24-s0001.xlsx Alpha diversity with metadata.

Table S2 msphere.00557-24-s0002.xlsx Abundance of genes detected in the metagenome of transgender males receiving testosterone therapy.

ASM does not own the copyrights to Supplemental Material that may be linked to, or accessed through, an article. The authors have granted ASM a non-exclusive, world-wide license to publish the Supplemental Material files. Please contact the corresponding author directly for reuse.
